# Combination of positron emission tomography/computed tomography and chest thin-layer high-resolution computed tomography for evaluation of pulmonary nodules: Correlation with imaging features, maximum standardized uptake value, and pathology

**DOI:** 10.1097/MD.0000000000011640

**Published:** 2018-08-03

**Authors:** Shasha Hou, Xiaoyun Lin, Shen Wang, Yiming Shen, Zhaowei Meng, Qiang Jia, Jian Tan

**Affiliations:** a Department of Nuclear Medicine, Tianjin Medical University General Hospital; b Graduate School, Tianjin Medical University, Tianjin, P.R. China.

**Keywords:** computed tomography, deoxyglucose, maximum standardized uptake value, positron emission tomography/computed tomography, pulmonary nodules

## Abstract

This study aimed to analyze the imaging findings of ^18^F-fluorodeoxyglucose positron emission tomography/computed tomography (^18^F-FDG PET/CT) and chest thin-layer high-resolution computed tomography (HRCT), correlate the maximum standardized uptake value (SUVmax), and the pathological type of benign or malignant pulmonary nodules (PNs), and assess the diagnostic accuracy in differentiating malignant from benign PNs.

A retrospective review of ^18^F-FDG PET/CT scans from 88 patients with PNs confirmed by pathology or clinical follow-up were included. They both accepted PET/CT and HRCT scan conventional. The final results were determined by a combination of PET/CT and HRCT. Independent samples *t* test was used for statistical analysis. Receiver operating curves (ROC) were generated and the optimal threshold of SUVmax was determined.

The sensitivity, specificity, and accuracy of HRCT, PET/CT, and PET/CT combined with HRCT in the diagnosis of PNs were 83.3%, 70%, 77.3%; 91.7%, 62.5%, 78.4%; and 95.8%, 75%, 86.4%, respectively. The SUVmax of malignant nodules was significantly higher than that of benign nodules, and the difference was statistically significant (*t* = –5.668, *P* < .001). In the subgroup analysis, the SUVmax of squamous cell carcinoma was higher than that of the denocarcinoma (*t* = –5.442, *P* < .001), and that of bronchioloalveolar carcinoma (*t* = 4.678, *P* < .001), the difference were both statistically significant. There were both no significant difference between adenocarcinoma and bronchioloalveolar carcinoma (*t* = 0.36, *P* = .722), tuberculosis and inflammatory nodules (*t* = –0.18, *P* = .858). Higher the value of SUVmax, greater the risk of malignancy. However, when the SUVmax ranges between 2.5 and 8.0, the lesion may be benign or malignant, and a comprehensive evaluation using combination methods with HRCT are required. When SUVmax <2.5, there is still a 9.5% chance of PN malignancy. ROC curve shows SUVmax >3.635 as the best threshold, and the sensitivity, specificity, accuracy, positive predictive value, and negative predictive value of PET/CT in diagnosis of PNs were 83.3%, 62.5%, 79.2%, 71.7%, and 71.4%, respectively.

PET/CT combined with HRCT should be advocated to improve the sensitivity, specificity, and accuracy of PET/CT in diagnosis of PNs.

## 1 Introduction

As part of the pulmonary nodules (PNs) lack the typical morphological characteristics, it is difficult to determine whether it is benign or malignant; thus, the differential diagnosis of PNs is always a clinical challenge. The new 2015 World Health Organization (WHO) classification of lung cancer provided the basis for a multidisciplinary approach emphasizing the close correlation among the clinical, radiologic, and molecular characteristics and histopathologic pattern of lung adenocarcinoma.^[1]^ Positron emission tomography/computed tomography (PET/CT) merges morphological and functional development techniques and is highly sensitive to tumor tissues, which is gradually applied to the diagnosis and differential diagnosis of PNs.^[2]^ In this study, the imaging of ^18^F-fluorodeoxyglucose positron emission tomography/computed tomography (^18^F-FDG PET/CT) in combination with chest thin-layer high-resolution computed tomography (HRCT), maximum standardized uptake value (SUVmax), and clinical data of 88 cases of PNs were analyzed retrospectively, with the intention to discuss the combined value of the 2 techniques in the differential diagnosis of PNs.

## 2 Material and methods

### 2.1 Study subjects

This retrospective study was performed at Tianjin Medical University General Hospital. This study was approved by the Ethics Committee of Tianjin Medical University General Hospital, and written informed consent was obtained from the patients.

We conducted a retrospective review of the ^18^F-FDG PET/CT database of our hospital and included 88 cases of PNs from patients who underwent ^18^F-FDG PET/CT imaging in our hospital from May 2015 to August 2017. The patient population included 60 men and 28 women (mean age, 61.2 ± 8.89 years; age range, 24–79 years). All the cases met the diagnostic criteria of pulmonary nodules and were confirmed by a clear pathologic diagnosis or clinical follow-up therapy. In all, there were 42 surgical cases, 12 underwent biopsy for bronchoscopy, 15 underwent thoracoscopy, 5 underwent percutaneous puncture under CT, and 14 cases were followed-up. Those whose lesions significantly reduced or were completely eliminated after clinical anti-inflammatory, antifungal, or anti-tuberculosis treatment were classified as benign lesions, and those whose lesions significantly enlarged and (or) showed significantly increased metabolic activity, metastasis, and caused death were diagnosed as malignant lesions. The operation was completed within a month after PET/CT imaging. Three of the followed-up patients died.

### 2.2 ^18^F-FDG PET/CT

All subjects were evaluated with FDG PET/CT for routine health check-ups, using an integrated Siemens Biograph mCT with 64 slice CT (Siemens Medical Solutions, Erlangen, Germany). Before the FDG injection, subjects were instructed to fast for at least 6 hours. Blood glucose level was measured and confirmed to be <140 mg/dL. Each subject was injected with an FDG dose of 5.55 MBq/kg. After the injection, patients underwent conventional PET/CT imaging after resting in silence for 45 to 60 minutes. For FDG PET/CT, a non-contrast CT was obtained first, after which an emission PET scan was performed from the skull base to the thigh. The PET images were acquired for 2 to 3 minutes scan/bed position with 3D mode. Then, PET images were reconstructed with 3.0-mm slice thickness using a 3D Ordered Subset Expectation Maximization (OSEM) iterative algorithm. All CT scan data were used for the attenuation correction of PET images. Image display and image fusion were conducted with axial, sagittal, and coronal positions. In addition, all patients underwent a chest thin layer high-resolution CT scan (HRCT).

### 2.3 Image analysis

The PET/CT data were reviewed by 2 experienced nuclear medicine physicians. The measurement of metabolic activity was accomplished with TrueD software (Siemens Medical Solutions). For SUVmax, region of interest (ROI) was automatically applied to each fusion PET/CT image, and the maximal standardized uptake value (SUVmax) was calculated to distinguish between benign and malignant PNs according to the edge of the nodules or the surrounding images in HRCT images. Further, the maximum diameter (dmax) of lesions in the cross-sectional images of HRCT lung window were measured (in mm).

#### 2.3.1 Diagnostic criteria for PET imaging

First, level of metabolism and the positive rate of the lesion in PET images were determined by taking the metabolic activity of the lesion higher than or equal to the mediastinal blood pool as positive and lower than the mediastinal blood pool as negative. The ROI was drawn in the CT slice, according to the edge of the lesion for those having no significant increase of metabolic activity and the SUVmax was determined on the corresponding PET slice.

#### 2.3.2 Diagnostic criteria for CT imaging

Diagnostic criteria for malignant PNs: lobulated sign, short speculated sign, pleural indentation, bronchial vessel convergence, vacuole sign, air bronchogram sign.

Diagnostic criteria for benign PNs: irregular shape, around thick strips, long spiculate sign, calcification in nodules and around satellite opacities, post pleura with a wide base, fuzzy edge.

#### 2.3.3 Diagnostic criteria for malignant PNs by PET/CT combined with HRCT

The final diagnosis was based on the combination of SUVmax, metabolic characteristics of PET/CT, and morphological signs of HRCT.

### 2.4 Statistical analysis

The Statistical Product and Service Solutions (SPSS 20.0) statistical software package was used for all statistical analysis. The SUVmax between the benign and malignant PNs of groups and subgroups were compared using the independent sample *t* test. *P* < .05 was considered to indicate statistical significance.

## 3 Results

A total of 88 subjects were included in the analysis. The baseline and PET/CT-HRCT-measured features of all the subjects are summarized in Table 1.

**Table 1:**
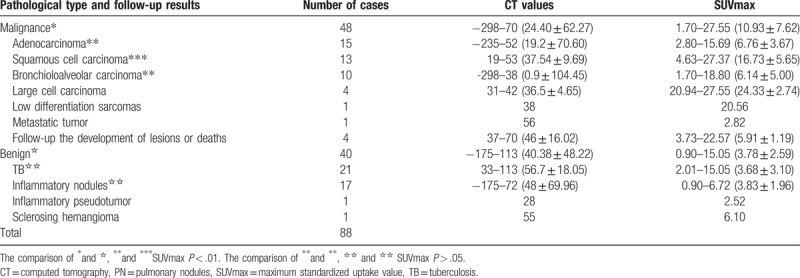
The CT value and SUVmax of benign and malignant PNs in different pathologic types (range, mean ± standard deviation).

### 3.1 Baseline characteristics of the nodules

The study included 88 cases of PNs, of which 48 were malignant nodules with a diameter range of 7 to 30 mm (mean: 2.13 ± 0.65 mm) and 40 were benign nodules with a diameter range of 10 to 30 mm (mean: 1.86 ± 0.62 mm).

Among these 88 subjects, there were 11 cases of subtuberous nodules of which 8 were malignant and 3 were benign. One case of ground glass nodules was an inflammatory tubercle.

### 3.2 The correlation analysis of SUVmax of benign and malignant PNs and different pathological types

In 88 cases of PNs, the SUVmax of malignant nodules (1.7–27.55; mean, 10.93 ± 7.62; n = 48) was significantly higher than that of benign nodules (0.9–15.05; mean, 3.78 ± 2.59; n = 40) (*t* = –5.668; *P* < .001). In the analysis of malignant nodular subgroups, the SUVmax of squamous cell carcinoma (4.63–27.37; mean, 16.73 ± 5.65; n = 13) was higher than that of adenocarcinoma (2.80–15.69; mean, 6.76 ± 3.67; n = 15) (*t* = –5.442; *P* < .001). The SUVmax 4.63 to 27.37 (mean 16.73 ± 5.65,n = 13) of squamous cell carcinoma was higher than the SUVmax 1.70 to 18.80 (mean 6.14 ± 5.00, n = 10) of bronchioloalveolar carcinoma, and it was statistically significant (*t* = 4.678, *P* < .001). There was no significant difference between the SUVmax values of adenocarcinoma and bronchial alveolar carcinoma (*t* = 0.36; *P* = .722). In the analysis of benign nodular groups, there was no statistically significant difference between the SUVmax values of tuberculosis (TB) and inflammatory nodules (2.01–15.05; mean, 3.68 ± 3.10; n = 21 vs 0.90–6.72; mean, 3.83 ± 1.96; n = 17, respectively) (*t* = –0.18; *P* = .858) (Table 1). Although the SUVmax range is wide, the mean value of malignant nodules is higher than the mean value of benign nodules.

Among all 88 cases, 67 cases showed an SUVmax >2.5 and included 47 malignant cases, 20 benign cases (1 case of inflammatory pseudotumor, SUVmax: 2.52), while 21 cases showed an SUVmax ≤2.5 and included 2 malignant cases (1 case each of highly differentiated adenocarcinoma and adenocarcinoma mainly based on bronchial alveolar carcinoma), 19 benign cases (including 12 cases of TB and 7 of inflammation). Generally, the chances of a nodule being malignant when SUVmax ≤2.5 is very slim, but our study shows a 9.5% chance of malignancy still exists. The results of this study found that if SUVmax was between 2.6 and 4.0 and/or 4.1 and 8.0, benign and malignant SUVmax would be overlapping, with a malignant chance of 53.8% and 53.6%, respectively,. When SUVmax >8.0, the malignancy chance was 96.2%. Generally, the higher the SUVmax, the greater the likelihood of malignancy (Table 2).

**Table 2:**
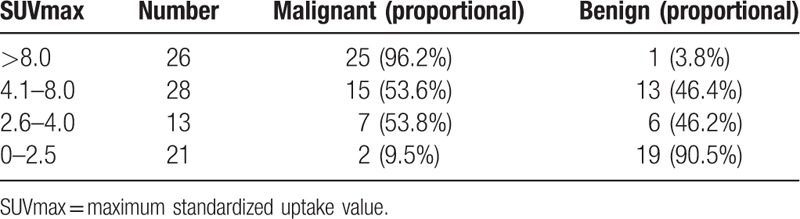
The correlation analysis of SUVmax and pathology of nodules.

#### 3.2.1 Analysis of diagnostic efficacy of PNs upon combination of PET/CT and HRCT

**Figure 1:**
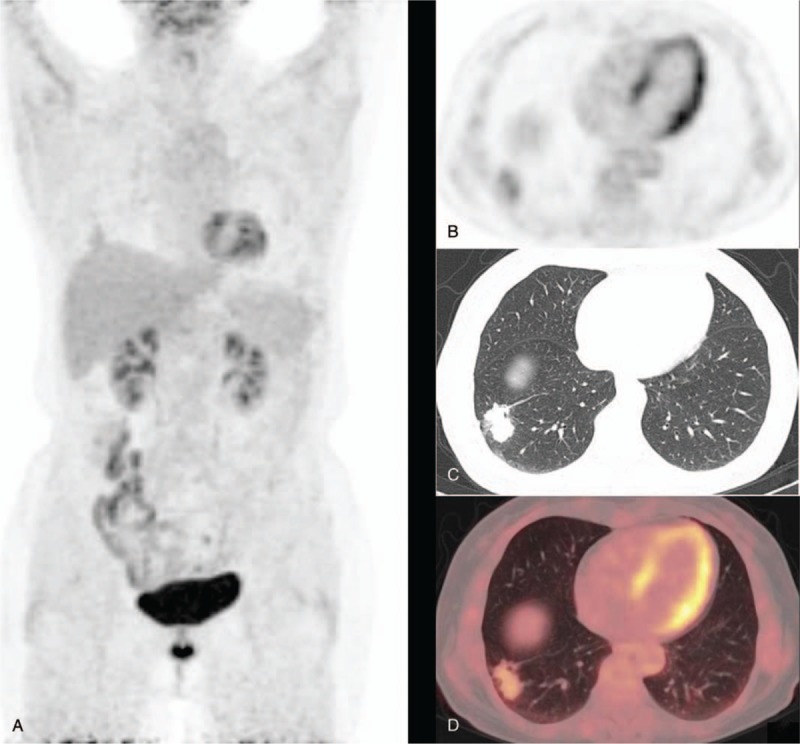
A 69-year-old female patient presented with a subsolid nodule outside the basal segment of the lower lobe of the right lung. The diameter of the nodule measured about 29 mm. The SUVmax and delayed SUVmax were 2.43 and 3.12, respectively. Histopathology revealed a highly differentiated adenocarcinoma. A shows PET MIP. B shows a PET image in transverse section. C shows a chest thin layer CT image. D shows a fusion image of PET/CT. PET/CT = positron emission tomography/computed tomography, SUVmax = maximum standardized uptake value. MIP = maximum intensity projection.

The diagnosis of malignant nodules by PET/CT combining with HRCT were obtained by the integration of the intaking images of PET/CT, SUVmax, and HRCT performance. Of 48 malignant lesions, 46 were correctly diagnosed by combining the two imaging techniques (14 adenocarcinoma, 9 bronchial alveolar carcinoma, 13 squamous carcinoma, 4 large cell carcinoma, 1 poorly differentiated sarcoma, 1 metastatic tumor, and 4 non-specific pathologic type); the remaining 2 cases were misdiagnosed. The PET/CT imaging of 46 nodules with correct diagnosis was characterized by an abnormal nodular radioactive concentration. The 2 cases of misdiagnosis were subtuberous nodules, whose pathologic types were highly differentiated adenocarcinoma and bronchial alveolar carcinoma, and their PET/CT image showed a concentration-like cloud (SUVmax: 1.70 and 2.43, respectively) (Fig. 1). HRCT showed that the lesion was characterized by an irregular shape and blurred edge and thick long ropes surrounding the edge with no typical malignant sign; these features likely resulted in misdiagnosis.

**Figure 2:**
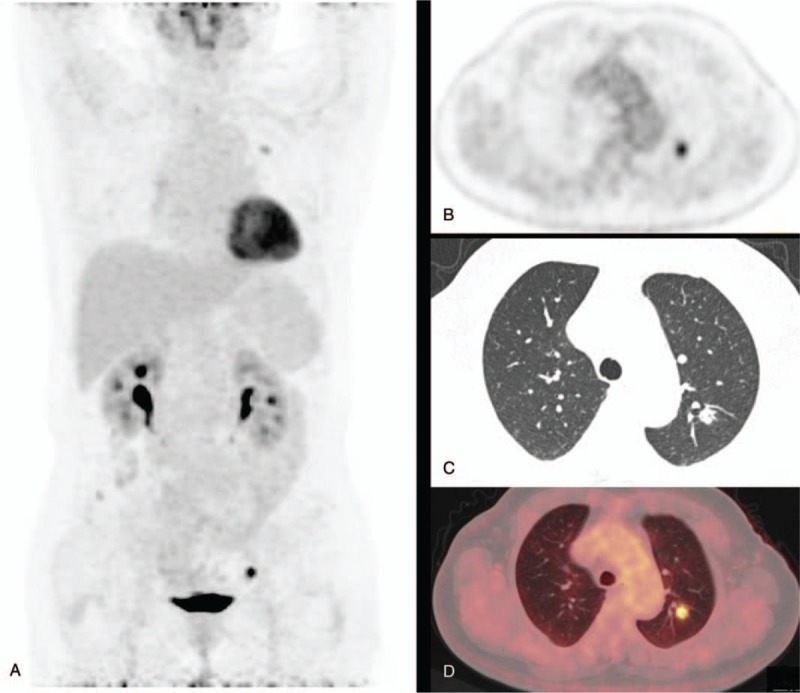
A 66-year-old female patient presented with an empty nodule with sharp edges at the posterior segment of the upper lobe of the left lung, whose diameter was about 13 mm and the SUVmax, 5.47. Histopathology revealed TB. A shows PET MIP. B shows PET image in transverse section. C shows chest thin layer CT image. D shows a fusion image of PET/CT. PET/CT = positron emission tomography/computed tomography, SUVmax = maximum standardized uptake value.

Of 40 benign lesions, 30 were correctly diagnosed by combining the 2 imaging techniques (13 inflammatory nodules, 16 TB, 1 sclerosing hemangioma), and 10 cases were misdiagnosed as malignant by both techniques. The HRCT imaging of the 30 correctly diagnosed nodules showed characteristics of benign lesions, namely, irregular shape, a fuzzy edge, surrounding thick strips, posting pleura with a wide base, calcification in nodules, and many small satellite opacities. Most of the nodules showed low uptake of ^18^F-FDG, with an SUVmax <2.5. Some of the nodules had high uptake, with an SUVmax >2.5, up to 6.08. The 10 misdiagnosed cases included 4 inflammatory nodules (SUVmax: 2.48–5.81), 1 inflammatory pseudotumor (SUVmax: 2.52), and 5 TB (SUVmax: 2.09–15.05) (Fig. 2). The PET/CT images of the 5 TB cases were characterized by circular nodular concentration, and the other 5 were characterized by irregular and patchy concentration. Because the simple HRCT images of all 10 cases were characterized by malignant signs at different levels, it may have resulted in misdiagnosis.

Of the 88 subjects, 77 were diagnosed correctly by the combination of uptake degree and form of PET/CT and HRCT imaging including 46 cases of malignance and 30 cases of benign. The sensitivity, specificity, and accuracy of the diagnosis of PNs by PET/CT in combination with HRCT were 95.8%, 75%, and 86.4%, respectively (Table 3).

**Table 3:**
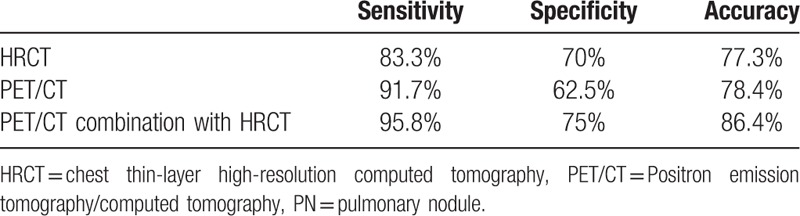
The efficiency of the diagnosis of PNs by HRCT, PET/CT, and PET/CT in combination with HRCT.

### 3.3 The analysis of ROC curve

**Figure 3:**
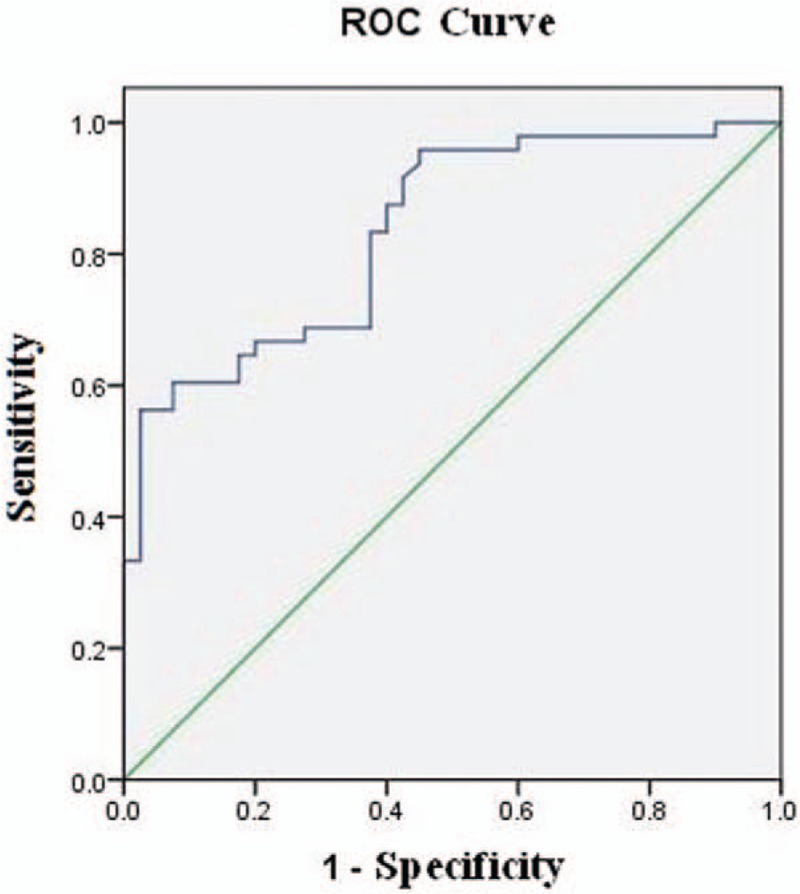
The ROC curve of the diagnosis of PN in a PET/CT mathematical prediction model (AUC = 0.835 ± 0.042). PET/CT = positron emission tomography/computed tomography, PN = pulmonary nodules, ROC = receiver operating curves.

After plotting the ROC curve of PN diagnosis in a PET/CT mathematical prediction model, the area under the curve was 0.835 ± 0.042 (Fig. 3). Taking SUVmax >3.635 as the optimal threshold, the area under the curve was 0.835 ± 0.042. The sensitivity, specificity, accuracy, positive predictive value, and negative predictive value of the diagnosis of PNs in a PET/CT mathematical prediction model were 83.3%, 62.5%, 79.2%, 71.7%, and 71.4%, respectively.

## 4 Discussion

Pulmonary nodules are space-occupying lesions in the lung that are single, spherical, or measuring <3 cm in diameter. With increasingly sophisticated imaging techniques and high-resolution CT, the detection rate of PNs is improving, with a 30% to 40% malignancy rate.^[3]^ Currently, PET/CT has been widely used in the diagnosis of isolated PNs. However, as the uptake of ^18^F-FDG is nonspecific for tumors, false positives and false negatives were also consistently found and proposed, and the diagnosis of PNs is no exception. Lesions assessed on PET/CT imaging are classified as benign or malignant by measuring the SUVmax of ^18^F-FDG and the morphological characteristics seen on PET/CT. Many benign lesions in the lung such as inflammatory, infectious, tuberculoid, and sarcoidosis can all cause increased metabolism and a subsequent increase in the concentration of ^18^F-FDG.^[4]^ Certain well-differentiated lung cancers, bronchioloalveolar carcinomas, and carcinoid tumors lead to false negative results owing to slow growth, high degree of differentiation, low metabolism, and less uptake of ^18^F-FDG.^[5,6]^

In this study, when the SUVmax was ≤2.5, there was still a 9.5% chance of the lesion being malignant, which was likely leading to false-negative PET/CT results. There were 2 cases of false negatives in simple PET/CT imaging (1 case each of well-differentiated adenocarcinoma and bronchioloalveolar carcinoma) and both were subsolid nodules. The diameter of the nodules was about 25 and 29 mm, respectively. The SUVmax was 2.43 and 1.70, respectively (Fig. 1). Lee and Lee^[7]^ suggested that the false negatives in PET/CT caused by pulmonary nonsolid malignant nodules mainly included atypical adenomatous hyperplasia, bronchioloalveolar adenocarcinoma, and adenocarcinoma mainly including bronchioloalveolar adenocarcinoma in terms of pathology, which was consistent with the results of our study. The uptake of ^18^F-FDG is mainly related to Glut-1 (facilitative glucose transporter), which is highly expressed in malignant tumor cells. A study^[8]^ found that Glut-l of some fine bronchioloalveolar carcinoma had excessive expression, but the expression was generally weak and not in the functional position, so the ^18^F-FDG could not be uptaken or resulted in very low intake. Moreover, the degree of differentiation of malignant tumors also affects the intake of ^18^F-FDG. In this study, 1 case of false negative was a highly differentiated adenocarcinoma, with a diameter of approximately 24 mm, and its low intake of ^18^F-FDG was likely to be associated with less tumor composition in the lesions. This study suggests that when SUVmax ≤2.5, the diagnosis of the highly differentiated lung cancer, fine bronchioloalveolar adenocarcinoma, and adenocarcinoma, which is mainly of bronchial alveoli in PET can be referenced. This should be simultaneously closely combined with the morphological features of HRCT, as having a detailed understanding of the size of the lesions in the dynamic phase change and clinical epidemiological data, increases the possibility of reducing misdiagnosis and improving the accuracy of diagnosis.

In addition, there were 2 cases of malignant subsolid nodules, including 1 case of fine bronchioloalveolar carcinoma, with an SUVmax as high as 8.75, and another case of unknown pathological type (due to lesions associated with transfer radioactive particles treatment rather than surgery) with an SUVmax of 3.74. The degree of uptake for ^18^F-FDG of the above 4 cases with subsolid nodules may be closely related to the degree of differentiation, but also to the size and mass of the tumor. In 2013, the Fleischner Society developed Guidelines for the Treatment of Pulmonary Non-solid Nodules that referred to the treatment of subsolid nodules and suggest that ^18^F-FDG PET/CT use should be considered, especially for the diameter of the solid nodules >10 mm.

Similarly, false positives also can affect the accuracy of diagnosis of PN in PET/CT. Inflammatory lesions and active TB are undoubtedly the common causes of the false-positive results in PET/CT imaging in benign nodules.^[9,10]^ In this study, when the SUVmax was between 2.5 and 8.0, the rate of benign lesions was about 46% because of the overlap of the benign SUVmax. The results of the study were related to the number of benign lesions and the degree of activity. Granulomatous nodules such as inflammatory and tuberculoid nodules include a high number of inflammatory cells such as macrophages, lymphocytes, and granulocytes that are very high in glucose metabolism, the expression of Glut-1 and Glut-3 and the transfer efficiency of glucose, and a subsequent increase in the uptake of ^18^F-FDG.^[8,11]^ When the obsolete pulmonary TB is in a stable condition, the TB lesions usually do not take up or take up very little of the ^18^F-FDG. The TB with high intake is often in a cell breeding period, because of a large number of these cells in the lesions. In this study, we had 3 cases of TB, 1 of inflammatory nodules, 1 of inflammatory pseudotumor, and 1 of organized pneumonia, with an SUVmax ranging between 2.09 and 15.05. The degree of metabolism appeared to have merged with that of malignant nodules, and as simple PET/CT images all characterized by circular nodular clusters, and simple HRCT also has different levels of malignancy signs, this could have led to misdiagnosis (Fig. 2). Therefore, when HRCT morphology, SUVmax, and images obtained by PET cannot distinguish the benign and malignant pulmonary nodules, laboratory tests such as Purified Protein derivative Tuberculin (PPD), Tuberculosis (TB) antibody, Tuberculosis infection t-cell spot test (T-SPOT), and Rapid detection of mycobacterium tuberculosis and rifampicin resistance (Xpert MTB/RIF) and treatment with anti-inflammatory agents may play a role in the differential diagnosis. At the same time, it is important to understand the dynamic changes in the size of the lesion to minimize misdiagnosis and improve diagnostic accuracy. When we retrospectively analyzed the misdiagnosed benign nodules, we found positive signs such as patchy calcifications of different degrees, long spiculate sign, surrounding satellite opacities in the edge of and in the lesions on careful observation of simple HRCT. Therefore, it is important to observe HRCT performance in the differential diagnosis of lung cancer, and careful resolution can correct diagnosis. This shows that the physician relying solely on PET/CT is likely to neglect the in-depth analysis of HRCT's performance in the lesion, which is one of the causes of misdiagnosis. Therefore, the physician responsible for disease diagnosis though PET/CT should be proficient in CT scans for malignant lesions of the lung and conduct a comprehensive analysis and judgement.

Although the SUVmax of benign and malignant lesions sometimes overlap, our study analyzed and compared the SUVmax between benign and malignant groups and found that the average SUVmax of malignant nodules was obviously higher than that of benign nodules, and the comparison of the 2 groups were statistically significant. We can see that although benign lesions also seem to have a slightly higher metabolic rate and even higher metabolic rate in the lungs in PET/CT,^[11,12]^ it is generally lower than the nodules of lung cancer. Subgroup analysis found that the average SUVmax of squamous cell carcinoma was higher than the average SUVmax of adenocarcinoma, and it was statistically significant. The study by de Geus-Oei et al showed that the intake of FDG of squamous cell carcinoma was significantly greater than that of adenocarcinoma, which is consistent with our results. Subgroup analysis also found that the SUVmax of large cell carcinomas and sarcomatoid carcinoma was significantly reduced than that of squamous cell carcinoma and adenocarcinoma. Because the number of cases was small, statistical comparisons were not done. However, it can be used as a reference for future clinical diagnosis. The SUVmax of TB compared with inflammatory nodules in benign nodules is not statistically significant, which may be related to the intensity of inflammatory response, extent of inflammation, and number of inflammatory cells.

The SUVmax is a significant influence on PET/CT in distinguishing benign and malignant PNs, but the diagnosis of benign and malignant lesions frequently overlaps. According to the results of the ROC curve, when the SUVmax >3.635, the sensitivity, specificity, accuracy, positive predictive value, and negative predictive value of the diagnosis of PNs by PET/CT were 83.3%, 62.5%, 79.2%, 71.7%, and 71.4%, respectively, and its diagnostic performance was lower than the results of PET/CT in combination with HRCT. This may be because of a selection bias in the inclusion of cases, and if the proportion of selected cases of malignant nodes was too high. There were more lesions with high-intake of FDG in benign nodules such as TB, inflammatory pseudotumor, pulmonary and cryptococcus, which leads to a large overlap of the SUVmax between benign and malignant nodules, and which is the reason for simple PET diagnosis being less effective.

PET/CT combined with HRCT has a high sensitivity of 95% to 97% and moderate specificity of 78% to 87% in the diagnosis of malignant nodules; thus, it is reliable for the identification of benign and malignant nodules.^[13]^ In YaLun's study,^[10]^ the sensitivity, specificity, and accuracy of PET/CT was about 96.7%, 75.7%, and 88.1%, respectively. In this study, the sensitivity, specificity, and accuracy of the diagnosis of malignant lung nodules of PET/CT combined with HRCT was 95.8%, 75%, and 86.4%, respectively, which were all higher than that of PET/CT and HRCT used alone, and the results were similar to the above study.

## 5 Conclusion

PET/CT is a kind of non-traumatic imaging examination method used in the differential diagnosis of benign and malignant lung nodules, whose uptake images of ^18^F-FDG, SUVmax, and HRCT manifestations of lesions should be carefully analyzed. Clinicians should avoid making a diagnosis based on only the metabolic rate in PET/CT images and SUVmax of lesions. Chest HRCT should be used more routinely and specifically in combination with laboratory tests and clinical epidemiological data to determine how to reduce false negative and false positive results of PNs diagnosis in PET/CT. When SUVmax <2.5, there is still a 9.5% chance for malignant and highly differentiated lung cancers; fine bronchioloalveolar adenocarcinoma should be taken into consideration. At the same time, a close follow-up should be conducted or a diagnosis combined with the pathology results should be made. When a pulmonary infection was suspected, the SUVmax was between 2.5 and 8.0, and the PET/CT and HRCT malignancies were atypical. In such cases, normal anti-inflammatory, anti-tuberculosis, or antifungal treatment should be recommended. After 2 weeks of treatment, the HRCT images were reviewed to eliminate infectious lesions and improve the rates of diagnosis. The diagnostic efficiency of the application of PET/CT combined with HRCT is higher than simple HRCT and PET/CT and it provides a better diagnostic method for the diagnosis and differential diagnosis of PN.

## Author contributions

**Conceptualization**: Jian Tan, Shasha Hou, Xiaoyun Lin

**Date curation:** Shasha Hou

**Formal analysis:** Shasha Hou, Xiaoyun Lin

**Investigation:** Shasha Hou, Xiaoyun Lin

**Methodology and Statistical Analysis:** Zhaowei Meng

**Project administration:** Jian Tan, Qiang Jia

**Software:** Shen Wang, Yiming Shen

**Visualization:** Jian Tan, Qiang Jia

**Writing – original draft:** Shasha Hou, Xiaoyun Lin

**Writing – review and draft:** Jian Tan
